# Common diagnostic/therapeutic errors in trigeminal autonomic cephalalgias and hemicrania continua: a systematic review

**DOI:** 10.1186/1129-2377-14-S1-P38

**Published:** 2013-02-21

**Authors:** M Viana, C Tassorelli, M Allena, G Nappi, F Antonaci

**Affiliations:** 1 Headache Science Center - C. Mondino National Institute of Neurology Foundation, IRCCS, Italy

## Introduction

Trigeminal autonomic cephalalgias (TACs) and Hemicrania Continua (HC) are relatively rare but clinically well-defined primary headaches. Despite the current clear-cut diagnostic criteria (2nd edition of the International Classification for Headache Disorders - ICHD-II) and several therapeutic guidelines, errors in work-up and treatment are frequently encountered in clinical practice.

## Objective

The aim of the present study is to investigate all published data dealing with mismanagement of patients affected by TACs and HC in order to understand and avoid the causes of such behaviors.

## Methods

We reviewed all the English language literature related to this particular topic.

## Results

The search strategy identified 65 published studies, 21 of which were relevant. The most frequent errors described in the management of patient with TACs and HC are the following: referral errors, diagnostic delay, misdiagnosis and the mismanagement using treatment without overt indication. Migraine with and without aura, trigeminal neuralgia, sinus infection, dental pain and temporomandibular dysfunction are the most frequently disorders overdiagnosed.

## Discussion

Although facing a clearcut clinical picture, TACs and HC are frequently not recognized and/or misdiagnosed with other disorders, not only by general physicians, but also by neurologists and headache specialists. This is mostly related to the limited knowledge of specific characteristics and variants of the disorders and it leads to the prescription of ineffective and sometimes invasive treatments, that may turn into heavy consequences on patients. Increasing the knowledge and the education concerning these disorders both in primary care physicians and expert in headache specialist could improve the quality of life of the patients suffering from TACs and HC.

## Competing interests

None.

## References

[B1] Van Alboom E Diagnostic and therapeutic trajectory of cluster headache patients in Flanders Acta Neurol Belg 2009 14 1 10 17 19402567

[B2] Rossi P Diagnostic delay and suboptimal management in a referral population with hemicrania continua Headache 2009 14 2 227 234 10.1111/j.1526-4610.2008.01260.x 19222596

